# Identification of oxidative stress-related biomarkers in uterine leiomyoma: a transcriptome-combined Mendelian randomization analysis

**DOI:** 10.3389/fendo.2024.1373011

**Published:** 2024-11-21

**Authors:** Yingxiao Li, Haoyue Chen, Hao Zhang, Zhaochen Lin, Liang Song, Chuanliang Zhao

**Affiliations:** ^1^ Department of Gynecology, The Affiliated Taian City Central Hospital of Qingdao University, Tai’an, Shandong, China; ^2^ Department of Rehabilitation Medical Center, The Affiliated Taian City Central Hospital of Qingdao University, Tai’an, Shandong, China; ^3^ Hydrogen Medical Research Center, The Affiliated Taian City Central Hospital of Qingdao University, Tai’an, Shandong, China; ^4^ Department of Orthopedics, The Affiliated Taian City Central Hospital of Qingdao University, Tai’an, Shandong, China; ^5^ Medical Integration and Practice Center, Shandong University School of Medicine, Shandong University, Jinan, Shandong, China

**Keywords:** uterine leiomyoma, oxidative stress, biomarkers, Mendelian randomization, transcriptome

## Abstract

**Background:**

Oxidative stress has been implicated in the pathogenesis of uterine leiomyoma (ULM) with an increasing incidence. This study aimed to identify potential oxidative stress-related biomarkers in ULM using transcriptome data integrated with Mendelian randomization (MR) analysis.

**Methods:**

Data from GSE64763 and GSE31699 in the Gene Expression Omnibus (GEO) were included in the analysis. Oxidative stress-related genes (OSRGs) were identified, and the intersection of differentially expressed genes (DEGs), Weighted Gene Co-expression Network Analysis (WGCNA) genes, and OSRGs was used to derive differentially expressed oxidative stress-related genes (DE-OSRGs). Biomarkers were subsequently identified *via* MR analysis, followed by Gene Set Enrichment Analysis (GSEA) and immune infiltration analysis. Nomograms, regulatory networks, and gene-drug interaction networks were constructed based on the identified biomarkers.

**Results:**

A total of 883 DEGs were identified between ULM and control samples, from which 42 DE-OSRGs were screened. MR analysis revealed four biomarkers: *ANXA1*, *CD36*, *MICB*, and *PRDX6*. Predictive nomograms were generated based on these biomarkers. *ANXA1*, *CD36*, and *MICB* were significantly enriched in chemokine signaling and other pathways. Notably, *ANXA1* showed strong associations with follicular helper T cells, resting mast cells, and M0 macrophages. *CD36* was positively correlated with resting mast cells, while *MICB* was negatively correlated with macrophages. Additionally, *ANXA1* displayed strong binding energy with amcinonide, and *MICB* with ribavirin.

**Conclusion:**

This study identified oxidative stress-related biomarkers (ANXA1, CD36, MICB, and PRDX6) in ULM through transcriptomic and MR analysis, providing valuable insights for ULM therapeutic research.

## 1 Introduction

Uterine leiomyoma (ULM), also referred to as uterine fibroids or myomas, represents the most prevalent benign tumor in the female reproductive system (1). Epidemiological evidence indicates that the incidence of ULM increases with age, with a particularly high prevalence among women of reproductive age. In women over 35 years, the incidence rate ranges from approximately 20% to 40% (2). Several factors, including environmental and psychological influences, contribute to ULM development. Notably, the elevation of estrogen receptor concentrations is widely accepted as a primary driver of ULM formation (3). Furthermore, familial clustering observed in numerous ULM cases suggests a substantial genetic component in its pathogenesis (4). Despite these insights, the precise molecular mechanisms underlying ULM remain incompletely understood, necessitating further investigation into potential pathogenic pathways and biomarkers to improve patient quality of life.

Oxidative stress refers to the disruption of the equilibrium between reactive oxygen species (ROS) production and the body’s endogenous antioxidant defenses (5). While basal levels of ROS are crucial for maintaining cellular homeostasis, excessive ROS can inflict structural and genetic damage, including protein and lipid peroxidation, which can impair cellular function and trigger apoptosis, contributing to disease progression (6). The uterus, within the female reproductive system, is particularly vulnerable to oxidative stress and subsequent DNA damage, which may be pivotal in ULM development (7, 8). Whole genome sequencing has elucidated the genetic foundation of ULM, revealing that over 70% of leiomyoma (LM) cases harbor mutations in the mediator complex subunit 12 (MED12), with mutation rates positively correlating with tumor quantity. ROS is implicated in both promoting MED12 mutations and facilitating tumor growth (9). Research suggests that uterine muscle contractions and vascular changes during the menstrual cycle induce hypoxic microenvironments within the uterine muscle, which subsequently trigger gene expression promoting leiomyoma proliferation under low oxygen conditions (10). Leiomyoma cells exhibit lower expression of antioxidant enzymes, such as superoxide dismutase (SOD) and catalase (CAT), compared to normal uterine muscle cells, particularly in hypoxic environments. ULM is characterized by elevated oxidative stress and insufficient antioxidant defense capacity (11). Therefore, investigating the role of oxidative stress in ULM pathogenesis is essential for advancing diagnostic and therapeutic strategies. Biomarkers of oxidative stress may emerge as valuable diagnostic tools for identifying patients with ULM. While medical intervention is often necessary for ULM treatment, antioxidants have shown potential in both prevention and therapeutic management, though further research is required. In summary, oxidative stress represents a critical and emerging aspect of ULM pathology, playing a central role in its progression. Further experimental and clinical research is needed to elucidate the cellular and molecular mechanisms linking oxidative stress to ULM.

Mendelian randomization (MR) represents a sophisticated approach in epidemiology and related fields for establishing causal relationships, utilizing genetic variation to evaluate the causal influence of potential risk factors on health outcomes. By leveraging the random assortment of alleles, MR effectively mitigates confounding factors that often obscure the exposure-outcome relationship in observational studies, enabling a more reliable and unbiased assessment of exposure effects on various outcomes compared to traditional methodologies (12, 13). MR analysis is contingent upon three fundamental assumptions: relevance (the genetic variant must be strongly associated with the exposure), independence (the genetic variant should be independent of confounders that affect the exposure-outcome relationship), and exclusion restriction (the genetic variant influences the outcome solely through its effect on the exposure, with no alternative direct causal pathways). Ensuring these assumptions are met is critical for the validity of causal inferences derived from MR studies (14). In parallel, the advent of microarray technology has revolutionized biological research by enabling high-throughput transcriptome analysis, transitioning investigations from single-gene studies to genome-wide expression profiling. Over the past two decades, microarray data have contributed substantially to advancing diverse areas of biological research, offering significant advantages for expression profiling and large-scale data analysis (15).

This study employed publicly available datasets, including GSE64763 and GSE31699, which are associated with ULM, alongside 436 oxidative stress-related genes. We chose GSE64763 because the dataset contains rich gene expression data covering multiple disease states and normal control samples, which is critical for identifying disease-associated genes and pathways. In addition, Dai Huang et al. (16) and Yumin Ke et al. (17) also used the GSE64763 dataset to conduct ULM-related research. The GSE31699 dataset, on the other hand, focuses on molecular marker studies of ULM, providing in-depth gene expression analysis, which is important for studying disease progression and treatment response mechanisms. Research by Lei Cai et al. (18) is based on this dataset. Genome-wide association study (GWAS) data for ULM and relevant candidate genes were sourced from the IEU OpenGWAS database. A comprehensive analytical framework was utilized, comprising differential expression analysis, weighted gene co-expression network analysis (WGCNA), MR, receiver operating characteristic (ROC) curves, and other statistical methodologies to identify oxidative stress-related biomarkers in ULM. Gene set enrichment analysis (GSEA) was performed to elucidate functional pathways associated with these biomarkers, while immune infiltration analysis was conducted to examine their role in the immune microenvironment. Additionally, potential therapeutic agents targeting the expression of these biomarkers were predicted (**Supplementary Figure S1**). Investigating the mechanisms underlying oxidative stress-related biomarkers in ULM is pivotal for advancing diagnostic and therapeutic strategies, as well as for guiding future research in this domain.

## 2 Materials and methods

### 2.1 Data source

The datasets GSE64763 and GSE31699 were extracted from the Gene Expression Omnibus (GEO) database (https://www.ncbi.nlm.nih.gov/geo/). GSE64763, used as the training set, contains transcriptomic microarray sequencing data from 25 uterine leiomyoma (ULM) samples and 29 control samples, based on the GPL571 sequencing platform (19). GSE31699, serving as the validation set, consists of 16 ULM and 16 control samples on the GPL6947 platform (20). A total of 436 oxidative stress-related genes (OSRGs) (21) were sourced from the Molecular Signatures Database (MSigDB) (https://www.gsea-msigdb.org/gsea/msigdb) (22), using the ‘msigdbr’ (v7.5.1) R package.

### 2.2 Weighted gene co-expression network analysis

To identify the module most strongly correlated with ULM, the R package ‘wgcna’ (v1.71) (23) was employed to perform WGCNA analysis on the GSE64763 samples. Initially, all samples were clustered to detect outliers, which were subsequently removed. Soft thresholding and scale-free network coefficients were selected to ensure the construction of a scale-free network. The module with the highest correlation to ULM was then identified.

### 2.3 Differential expression analysis and functional enrichment analysis

The differentially expressed genes (DEGs) between ULM and normal samples were identified using the ‘limma’ (v3.50.1) R package (24), applying the cut-off criteria of adj.p.value < 0.05 and |log2(FoldChange)| > 0.5. The DEGs were visualized using a volcano plot with the ‘ggplot2’ package (v3.4.1) (25), and a heatmap was generated with the ‘ComplexHeatmap’ package (v2.14.0) (26). Next, upregulated DEGs were intersected with genes from the ULM module positively correlated with ULM, while downregulated DEGs were intersected with negatively correlated ULM module genes. And these two sets of intersecting genes were combined to obtain the ULM-associated genes, which were intersected with 436 OSRGs to yield the differentially expressed OSRGs (DE-OSRGs). Subsequently, gene ontology (GO) and kyoto encyclopedia of genes and genomes (KEGG) enrichment analyses based on GO and KEGG databases (https://www.geneontology.org, https://www.genome.jp/kegg/) were performed using the ‘clusterProfiler’ (v4.2.2) R package (27) to explore the biological functions and pathways associated with these candidate genes.

### 2.4 Mendelian randomization analysis

MR analysis was conducted to investigate the causal relationship between the exposure factors (DE-OSRGs) and the outcome (ULM). Using the extract_instruments function of the R package ‘TwoSampleMR’ (v0.5.6) (28), we read the data related to the exposure factors and screened the instrumental variables (SNPs) according to the following criteria: *p* = 5*10^^-8^ to ensure that the instrumental variables were significantly correlated with the exposure factors; Enable clump = TRUE to exclude the effect of linkage disequilibrium (LD); set r^2^ = 0.001 and kb = 200. Next, the extract_outcome_data function was used to read the outcome data and filter out instrumental variables that were associated with exposure factors but not with the outcome by setting proxies = TRUE and rsq = 0.8. In addition, we calculated the F-statistics of the instrumental variables, and when F < 10, it indicated that the instrumental variables were weak instrumental variables and needed to be excluded. The above correlation analysis and the removal of weak instrumental variables ensured that these instrumental variables satisfied the correlation assumptions. Subsequently, five univariate MR methods were employed: MR Egger, Weighted Median, Inverse Variance Weighted (IVW) (29), Simple Mode, and Weighted Mode (30). The IVW method is based on Mendelian laws of inheritance, which ensure the random distribution of locus alleles in the population. This property makes genetic variants (e.g., SNPs) ideal instrumental variables for probing causal associations between exposures and diseases. IVW is effective in reducing confounding factors and reverse causality bias in traditional observational studies, and has evolved over the years into a mature and reliable method for causal inference. Therefore, IVW has become our primary method for identifying biomarkers causally associated with ULM. A *p*-value less than 0.05 was considered evidence of a causal association. A scatter plot was used to assess the relationship between exposure factors and outcomes, while a forest plot identified the predictive exposure factors for each single nucleotide polymorphism (SNP) in relation to the outcome. Finally, a funnel plot was utilized to evaluate whether the MR analysis adhered to Mendel’s second law of randomization.

### 2.5 Sensitivity analysis

To assess the robustness of the MR results, a sensitivity analysis was performed. Firstly, the heterogeneity analysis was carried out by using the mr_heterogeneity function in the R package ‘TwoSampleMR’ (v0.5.6) (28), and if the Q *p*-value was lower than 0.05, it indicated the existence of heterogeneity. Next, the horizontal polytropy test was performed by the mr_pleiotropy_test function, and if the *p*-value was greater than 0.05, it indicated that there were no confounders. Finally, the mr_leaveoneout function was used to perform a leave-one-out (LOO) analysis (step-by-step culling of each SNP and calculation of the meta effect of the remaining SNPs) and the inverse variance weighted method was used to assess the changes in the results, which were visualized by forest plots. The exclusivity assumption was satisfied by sensitivity analyses that ensured the genetic variation influenced outcomes only through exposure factors, excluding the role of other potential pathways. At the same time, the independence assumption was satisfied by excluding instrumental variables associated with confounders. In addition, the proportion of variance explained may also affect the validity of the MR analysis. In general, a higher proportion of variance explained implies stronger instrumental variables, which improves the confidence of the results. If SNPs explain only a small amount of variance, this may lead to bias and invalid causal inferences.

### 2.6 Receiver operating characteristic curve

To evaluate the diagnostic potential of the identified biomarkers for ULM, an ROC analysis was performed for each individual gene using the R package ‘pROC’ (v1.18.0) (31). The diagnostic accuracy increased as the area under the curve (AUC) approached 1, while an AUC of 0.5 indicated no diagnostic value.

### 2.7 Establishment of alignment diagram

Based on biomarker expression, an alignment diagram was constructed using the ‘rms’ R package (32). The predictive accuracy of this diagram was assessed using a calibration curve, decision curve analysis (DCA), and clinical impact curve (CIC).

### 2.8 Functional similarity and correlation analysis of biomarkers

To investigate the functional similarities among biomarkers, functional annotations were carried out with the ‘GOSemSim’ R package (v2.24.0) (33), focusing on three aspects: biological processes (BP), cellular components (CC), and molecular functions (MF). The average similarity scores of genes at different levels were calculated, and biomarkers were ranked based on these average similarity values. Additionally, Spearman correlation analysis of biomarkers was conducted using the ‘corrplot’ R package (v0.92) (34).

### 2.9 Gene set enrichment analysis

First, the correlation coefficients between each biomarker and other genes were calculated, after which genes were ranked according to these coefficients, yielding a list of genes relevant to each biomarker. The C2: KEGG gene sets in the MSigDB database served as the reference gene sets for GSEA enrichment analysis.

### 2.10 Immunoinfiltration analysis

To examine the relationship between biomarkers and immune cells, the abundance of 22 types of immune-infiltrating cells in the training set samples was estimated. Wilcoxon tests were then applied to calculate differences in immune cell infiltration between the ULM and control groups, identifying differential immune-infiltrating cells. Spearman correlation analysis was conducted to evaluate the relationships between differential immune-infiltrating cells, and between the biomarkers and the differential immune-infiltrating cells.

### 2.11 Regulatory network

The Network Analyst platform (https://www.networkanalyst.ca/) was utilized to predict transcription factors (TFs) regulating the identified biomarkers. A TF-mRNA regulatory network was constructed using Cytoscape. Additionally, to explore the regulatory mechanisms of biomarkers in ULM, corresponding miRNAs were predicted using the TarBase (http://www.microrna.gr/tarbase) and miRTarBase (https://mirtarbase.cuhk.edu.cn) databases. The miRNA-mRNA relationship pairs obtained from both databases were integrated, and miRNet was then used to predict lncRNAs corresponding to the miRNAs. Ultimately, a lncRNA-miRNA-mRNA regulatory network was established.

### 2.12 Disease and drug analysis

To assess the role of biomarkers in other uterine diseases, their relationships were analyzed through the CTD database (https://ctdbase.org). The DGidb database (https://dgidb.org) was then employed to predict small-molecule drugs associated with the biomarkers, and a gene-drug interaction network was constructed. Protein structures of biomarkers were retrieved from the PDB database (https://www1.rcsb.org/), while 3D structures of the therapeutic compounds were sourced from the NCBI PubChem Compound database (https://www.ncbi.nlm.nih.gov/pccompound/). Molecular docking was performed using CB-Dock to identify the best binding conformations between protein targets and drugs. Pymol was used for the visualization of these interactions. Binding energies below -5 kcal/mol were considered indicative of strong binding affinity between the compound and its target.

### 2.13 Expression and validation of biomarkers

The expression levels of the biomarkers were assessed in both ULM and control samples. To further verify the accuracy of these results, the biomarkers were validated in the independent validation set.

### 2.14 Real-time quantitative polymerase chain reaction

A total of five paired normal and ULM samples were collected from The Affiliated Taian City Central Hospital of Qingdao University. All participants provided informed consent, and the study received approval from the hospital’s ethics committee.

Total RNA was extracted from the 10 samples using TRIzol reagent (Invitrogen, China), following the manufacturer’s protocol. RNA concentrations were measured using the NanoPhotometer N50. cDNA was synthesized through reverse transcription using the SureScript First-strand cDNA synthesis kit (Servicebio, China). RT-qPCR was performed on the CFX Connect Thermal Cycler (Bio-Rad, USA), and relative mRNA expression levels were quantified using the 2-^ΔΔCT^ method. The sequences of all primers are available in **Supplementary Table 1**.

### 2.15 Statistical analysis

The data underwent processing and analysis utilizing R software (v4.2) (35). The Wilcoxon test was used for comparison between groups, and statistical significance was assessed based on a *p*-value below 0.05.

## 3 Results

### 3.1 Identification of key module genes associated with ULM

**Figure 1:**
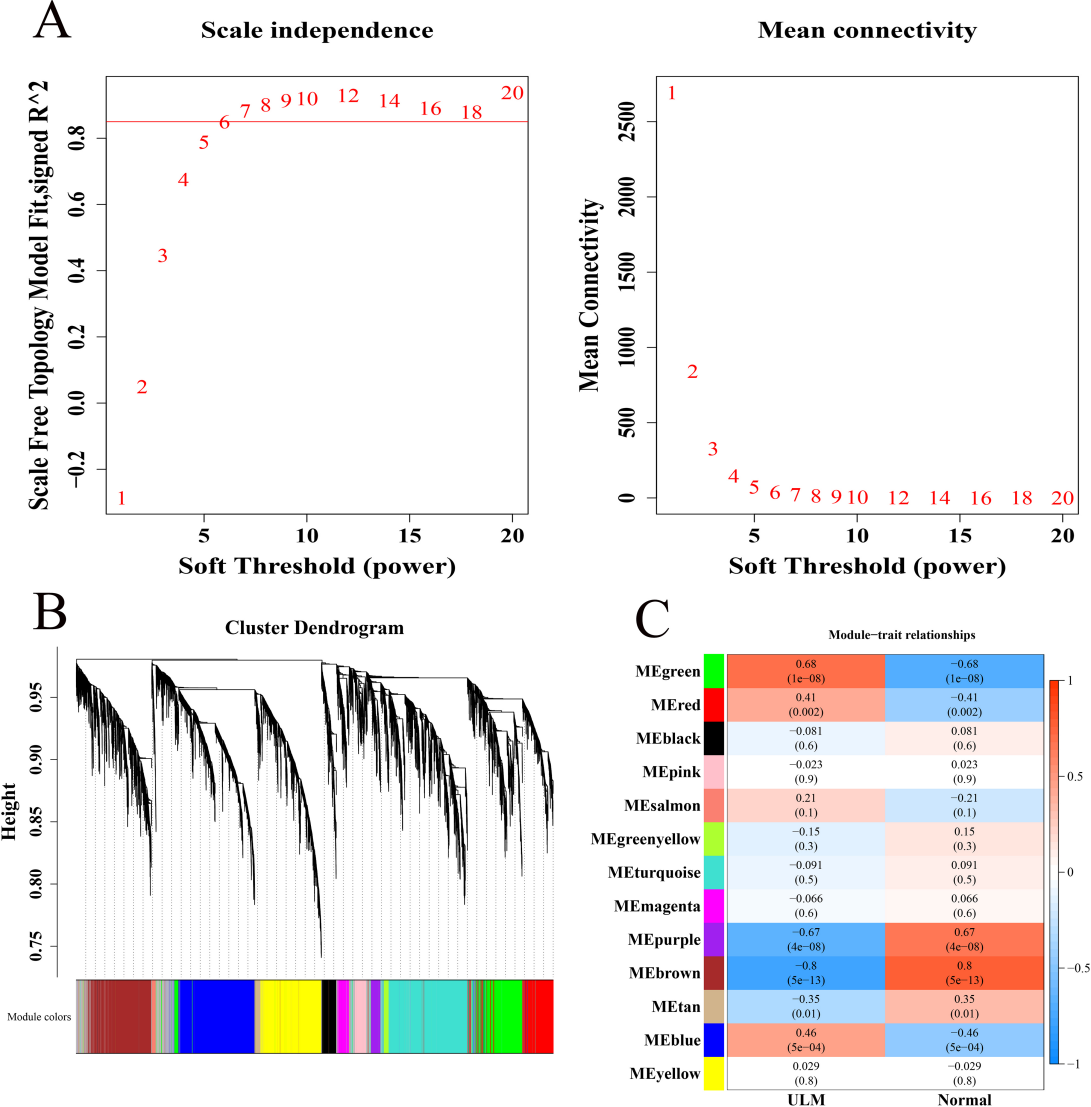
Identification of key module genes associated with ULM. **(A)** Soft threshold filtering. **(B)** Gene dendrogram and modules before merging. **(C)** Heatmap of module correlations with phenotypes.

In the GSE64763 dataset, all samples were clustered without notable outliers (**Supplementary Figure 2**). With R^2^ set to 0.85, the soft threshold β was determined to be 7 (**Figure 1A**), enabling the construction of a scale-free network. Thirteen co-expression modules were identified through WGCNA (**Figure 1B**). Among these, the MEgreen module (cor = 0.68, 1305 genes) exhibited a strong positive correlation with ULM, while the MEbrown (cor = -0.8, 1724 genes) and MEpurple (cor = -0.67, 261 genes) modules showed strong negative correlations with ULM, marking them as key ULM-related modules (**Figure 1C**).

### 3.2 Differential gene expression analysis and functional enrichment analysis of DE-OSRGs

**Figure 2:**
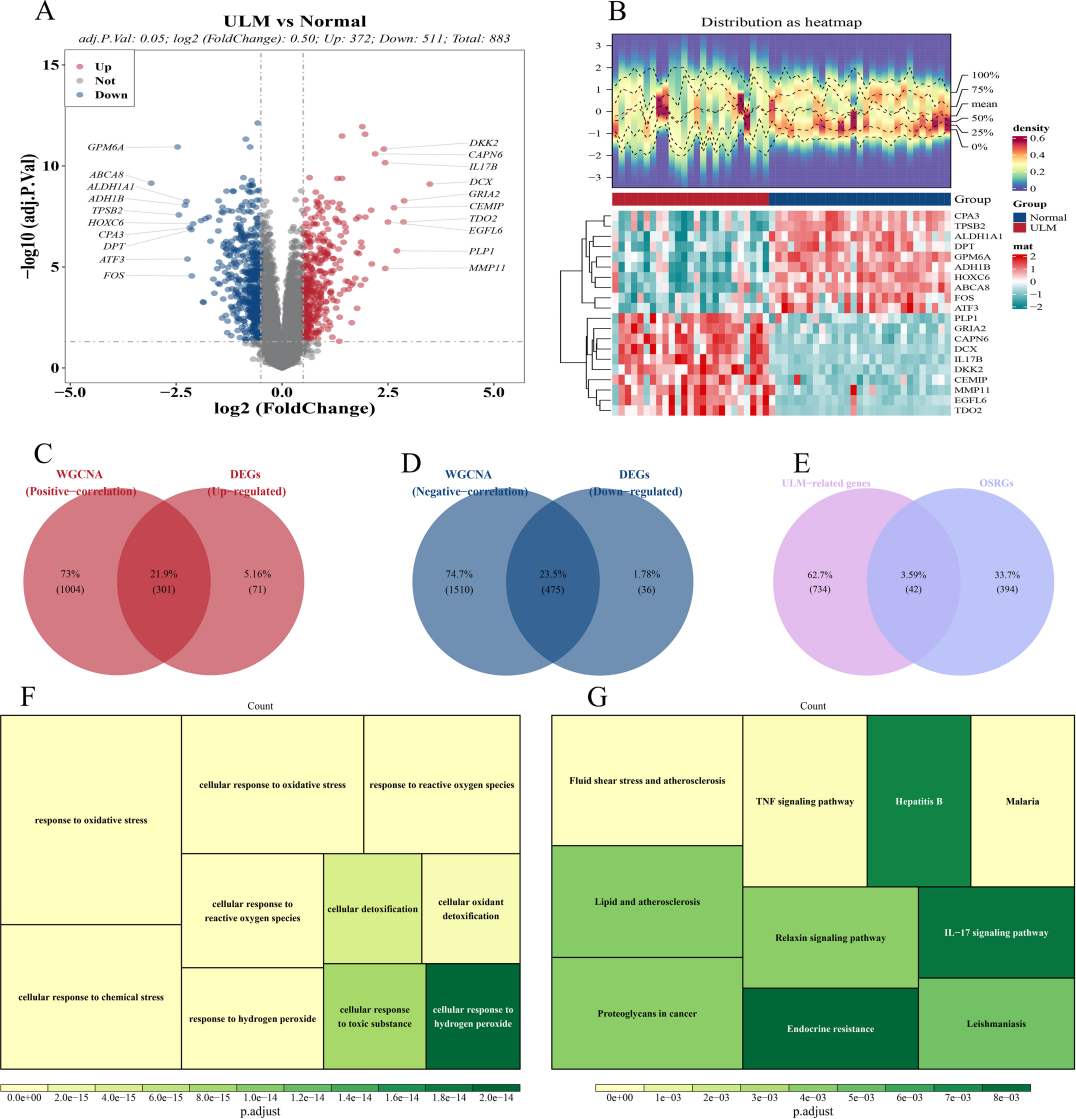
Differential expression analysis and enrichment analysis. **(A)** Volcano plot of DEGs. **(B)** Heatmap of DEGs. **(C)** Venn diagram showing 301 intersection genes from up-regulated DEGs and ULM positively correlated module genes. **(D)** Venn diagram showing 475 intersection genes from down-regulated DEGs and ULM negatively correlated module genes. **(E)** Venn diagram showing 42 DE-OSRGs from the intersection of 776 ULM-related genes and 436 OSRGs. **(F)** GO analysis of DE-OSRGs. **(G)** KEGG pathway analysis of DE-OSRGs.

In the GSE64763 dataset, 883 DEGs were identified between ULM and control samples, consisting of 372 upregulated and 511 downregulated genes (**Figures 2A, B**). Subsequently, 776 ULM-related genes were identified, including 301 upregulated genes positively correlated with ULM (**Figure 2C**) and 475 downregulated genes negatively correlated with ULM (**Figure 2D**). Through the intersection of 776 ULM-related genes and 436 OSRGs, 42 DE-OSRGs were identified (**Figure 2E**). GO analysis indicated that these DE-OSRGs were predominantly involved in cellular response to hydrogen peroxide, cellular response to toxic substance, cellular detoxification, response to oxidative stress, cellular response to oxidative stress, response to reactive oxygen species, cellular response to chemical stress, cellular response to reactive oxygen species, response to hydrogen peroxide and cellular oxidant detoxification pathways (**Figure 2F**). KEGG pathway analysis revealed enrichment in the IL-17 signaling pathway, endocrine resistance, hepatitis B, lipid and atherosclerosis, proteoglycans in cancer, relaxin signaling pathway, leishmaniasis, fluid shear stress and atherosclerosis, malaria, and TNF signaling pathway (**Figure 2G**).

### 3.3 Identification of four biomarkers through MR analysis

**Figure 3:**
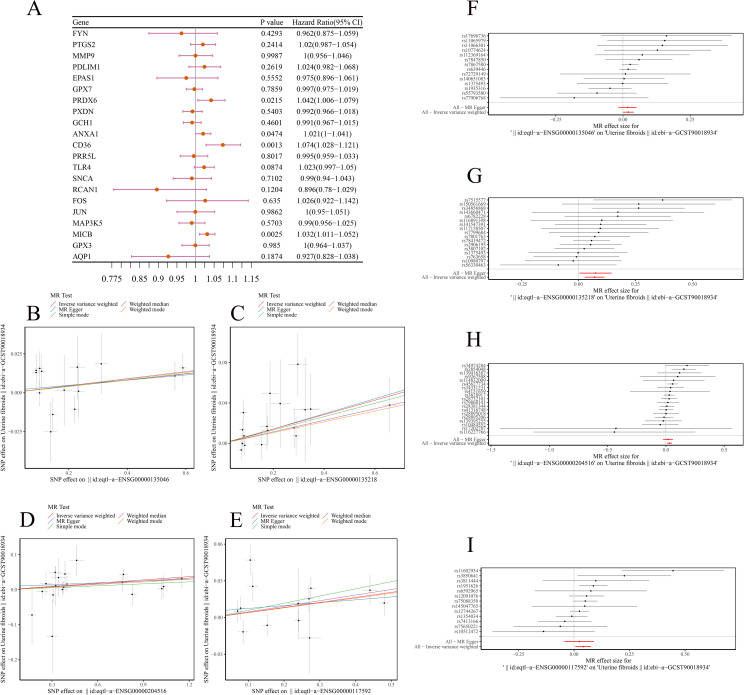
MR analysis. **(A)** Forest plot showing MR analysis of ULM candidate genes. **(B–E)** Scatter plots showing positive causal relationships of *ANXA1*, *CD36*, *MICB*, and *PRDX6* with ULM. **(F–I)** Forest plots predicting SNP loci exposure factors for outcome diagnosis.

**Figure 4:**
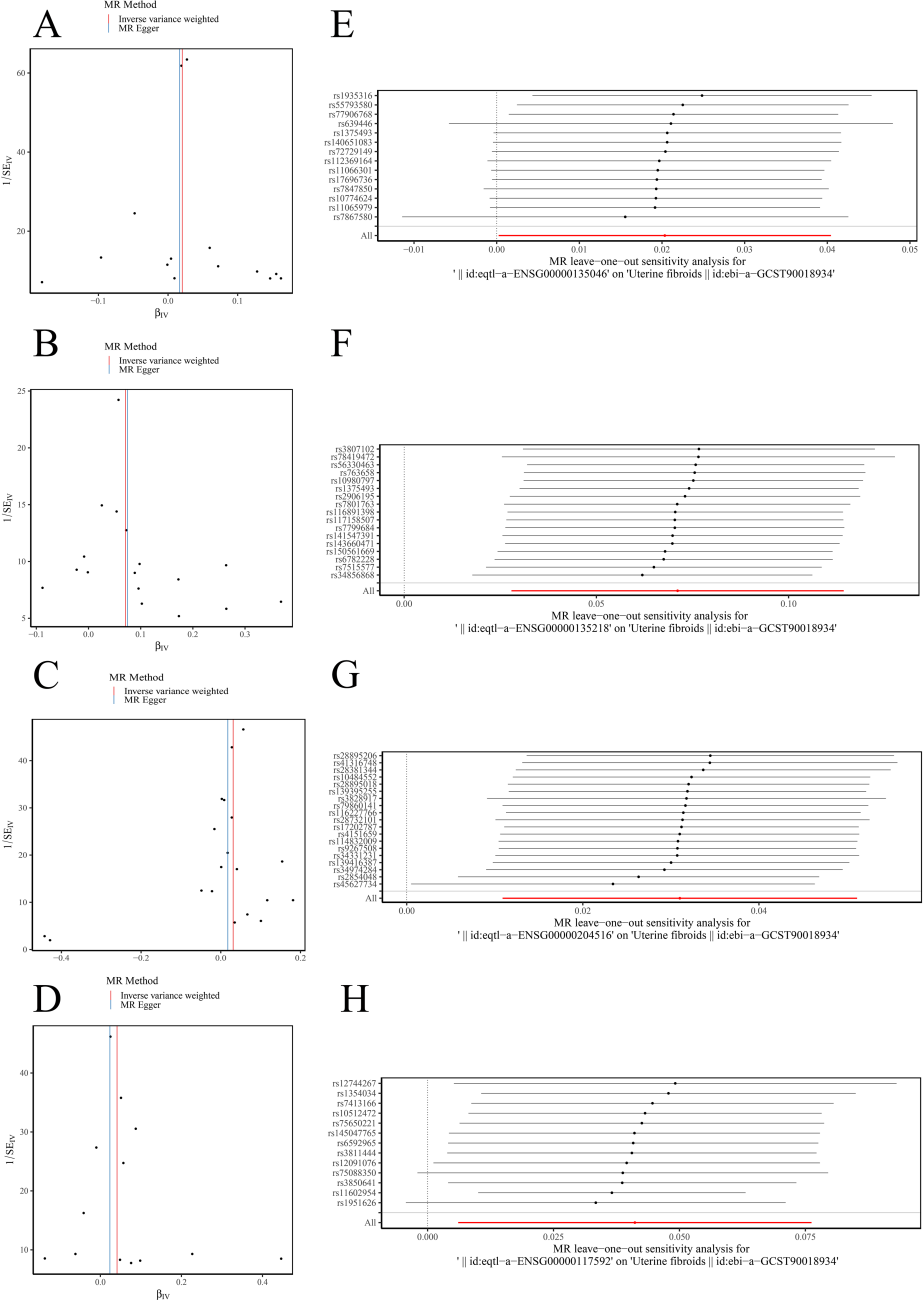
Sensitivity analysis for MR results. **(A–D)** Funnel plots showing uniform SNP distributions for biomarkers *ANXA1*, *CD36*, *MICB*, and *PRDX6*. **(E–H)** Leave-one-out analysis for *ANXA1*, *CD36*, *MICB*, and *PRDX6*.

MR analysis was conducted to assess the relationship between DE-OSRGs and ULM. F-statistics for SNP were shown in **Supplementary Table S2**. Based on the IVW method, four biomarkers—*ANXA1* (*p* = 0.0474, odds ratio (OR) = 1.021), *CD36* (*p* = 0.0013, OR = 1.074), *MICB* (*p* = 0.0025, OR = 1.032), and *PRDX6* (*p* = 0.0215, OR = 1.042)—demonstrated significant causal relationships with ULM, all with *p* < 0.05. The OR values greater than 1 suggest these biomarkers are potential risk factors for ULM (**Figure 3A**). Scatter plots for the five algorithms showed positive slopes, confirming consistent and robust positive causal relationships with ULM (**Figures 3B–E**). Moreover, the MR effect sizes in the forest plots were all above 0, further supporting these biomarkers as ULM risk factors (**Figures 3F–I**). The funnel plot revealed uniform SNP distributions for *ANXA1*, *CD36*, *MICB*, and *PRDX6*, indicating consistency with Mendel’s second law of random assortment (**Figures 4A–D**).

Sensitivity analysis revealed that the heterogeneity (**Table 1**) and pleiotropy (**Table 2**) tests for *ANXA1*, *CD36*, and *MICB* yielded *p*-values greater than 0.05, suggesting no significant heterogeneity or horizontal pleiotropy. Although the *p*-value for the heterogeneity test of PRDX6 was 0.0153, the IVW method remained unaffected, ensuring reliable MR results. The leave-one-out sensitivity tests further demonstrated that the SNPs for the exposure factors and outcomes were consistently aligned to the right of 0, with no substantial deviations, confirming stable results without excessive sensitivity to individual SNPs (**Figures 4E–H**). Therefore, *ANXA1*, *CD36*, *MICB*, and *PRDX6* were confirmed as reliable biomarkers for ULM.

**Table 1:** MR heterogeneity test.

Gene	Method	Q	Q_df	Q_pval
*ANXA1*	Inverse variance weighted	13.3013	13	0.4248
*CD36*	Inverse variance weighted	13.6213	16	0.6269
*MICB*	Inverse variance weighted	17.3819	18	0.4970
*PRDX6*	Inverse variance weighted	24.9094	12	0.0153

**Table 2:** MR pleiotropy test.

Gene	egger_intercept	se	pval
*ANXA1*	0.0014	0.0053	0.7898
*CD36*	-0.0007	0.0056	0.8983
*MICB*	0.0089	0.0129	0.4964
*PRDX6*	0.0051	0.0083	0.5514

### 3.4 Diagnostic value of biomarkers

**Figure 5:**
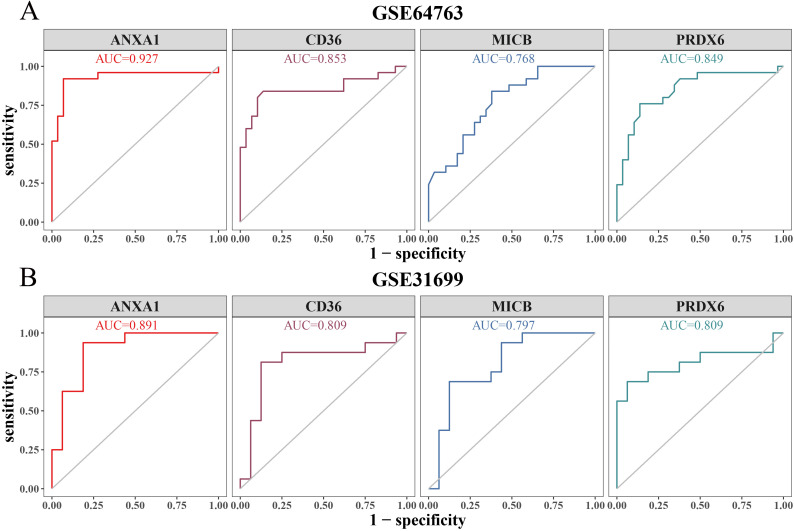
ROC curve analysis. **(A)** ROC curve of biomarkers in the training set (GSE64764). **(B)** ROC curve of biomarkers in the validation set (GSE31699).

In the GSE64763 dataset, the AUC values for *ANXA1*, *CD36*, *MICB*, and *PRDX6* were 0.927, 0.853, 0.768, and 0.849, respectively (**Figure 5A**). Similarly, in the GSE31699 dataset, all biomarkers exhibited AUC values exceeding 0.7 (**Figure 5B**), confirming their diagnostic potential.

### 3.5 Establishment and validation of a predictive alignment chart

**Figure 6:**
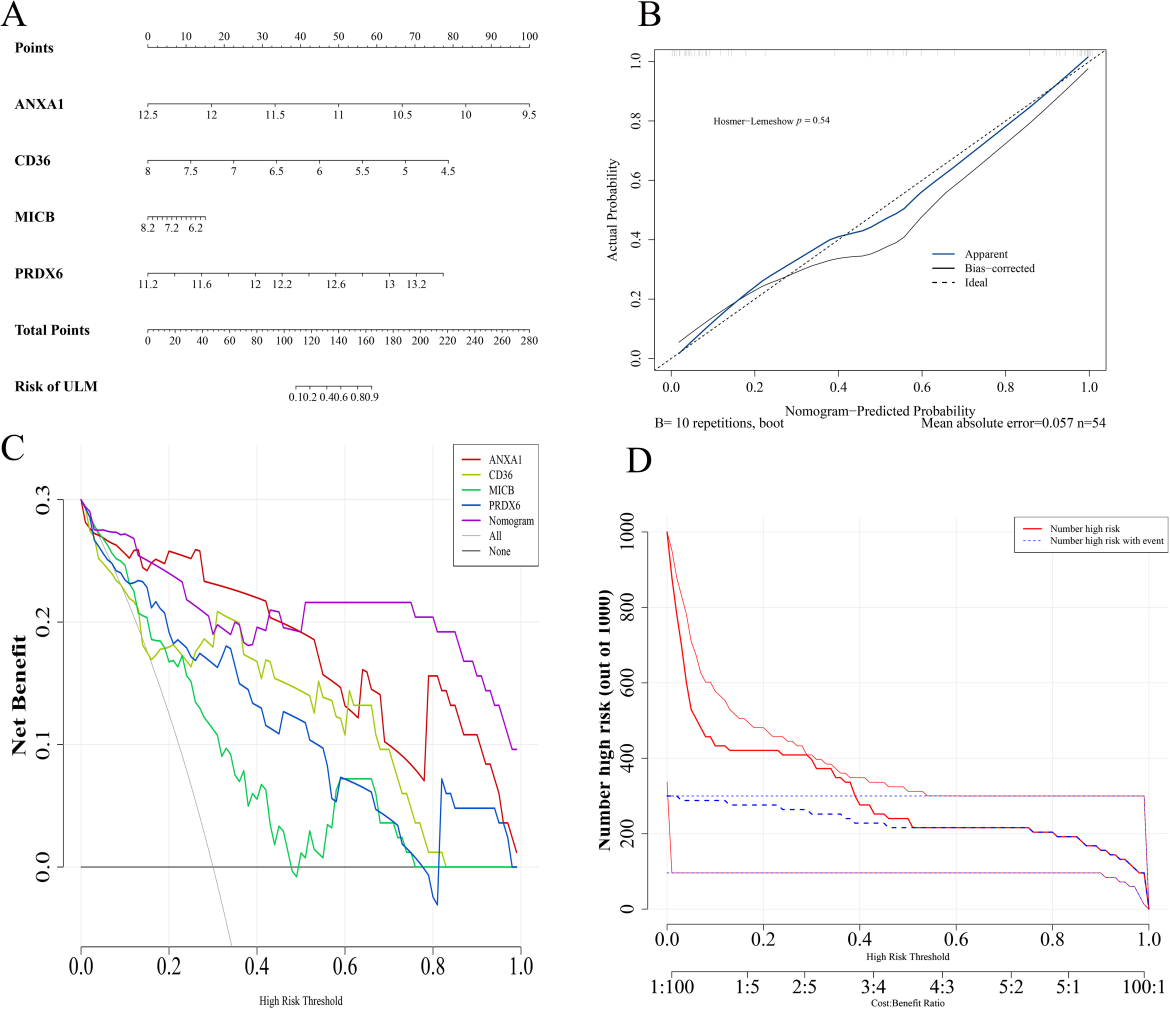
Biomarker alignment diagram. **(A)** Alignment diagram model based on biomarkers. **(B)** Calibration curves. **(C)** DCA curves. **(D)** Clinical impact curves evaluating the alignment diagram’s predictive power.

To enhance predictive efficiency, these biomarkers were used to construct a predictive alignment chart in the training set (**Figure 6A**). The *p*-value from the Hosmer-Lemeshow test was 0.54, indicating no significant difference between predicted and ideal values (**Figure 6B**). The DCA demonstrated that the alignment chart had a graceful benefit rate, underscoring its refined predictive capability (**Figure 6C**). CIC analysis further evaluated the clinical utility of the alignment chart, revealing a favorable net benefit across a wide and practical threshold probability range, suggesting the chart’s significant predictive value (**Figure 6D**).

### 3.6 Correlation and pathway analysis of biomarkers

**Figure 7:**
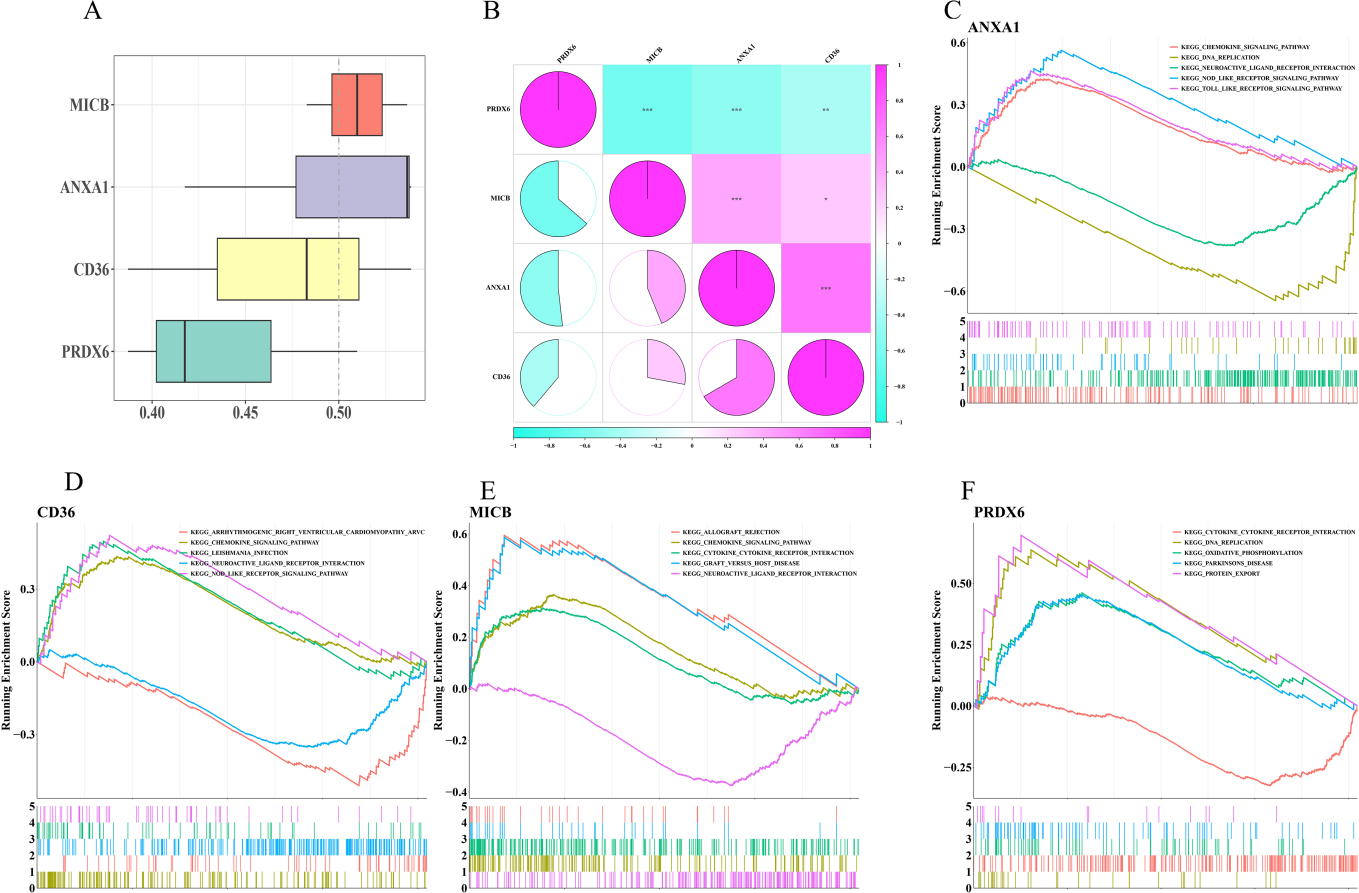
Correlation and pathway analysis of biomarkers. **(A)** Functional similarity analysis of biomarkers. **(B)** Heatmap of biomarker correlations. **(C–F)** GSEA for *ANXA1*, *CD36*, *MICB*, and *PRDX6*.

To explore biomarker correlations, GO analysis indicated that *MICB* and *ANXA1* shared higher average functional similarity (**Figure 7A**). *CD36* and *ANXA1* exhibited the strongest positive correlation with a coefficient of 0.666, while *MICB* and *PRDX6* displayed the strongest negative correlation (cor = -0.636) (**Figure 7B**). Additionally, GSEA revealed that *ANXA1*, *CD36*, and *MICB* were significantly enriched in chemokine signaling pathways and neural active ligand-receptor interactions (**Figures 7C–E**), while *PRDX6* was prominently enriched in oxidative phosphorylation and cytokine-cytokine receptor interactions (**Figure 7F**).

### 3.7 Correlation between immune infiltration and biomarkers

**Figure 8:**
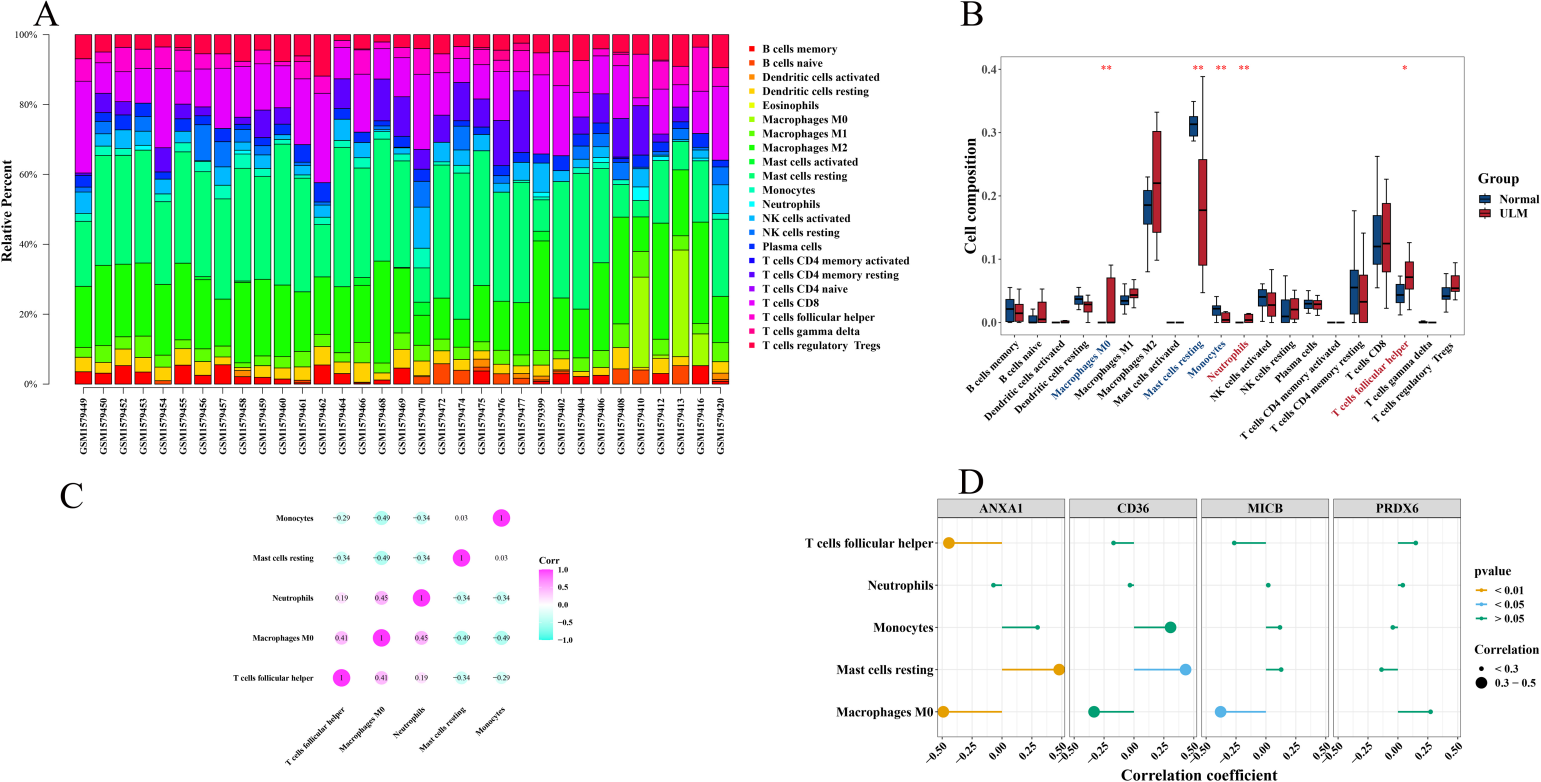
Immune infiltration correlation with biomarkers. **(A)** Immune cell infiltration abundance in the training set (GSE64763). **(B)** Boxplots comparing differential immune-infiltrating cell abundance between ULM and normal. **(C)** Heatmap of correlations between differentially immune-infiltrating cells. **(D)** Lollipop plot showing correlations between immune-infiltrating cells and biomarkers. *p < 0.05; **p < 0.01.

The relationship between biomarkers and immune infiltration was investigated by calculating the abundance of 22 types of immune-infiltrating cells (**Figure 8A**). Notably, follicular helper T cells and neutrophils were significantly more abundant in ULM samples compared to controls, while M0 macrophages, resting mast cells, and monocytes were more prevalent in the control group (**Figure 8B**). Spearman correlation analysis revealed a strong positive association between M0 macrophages and neutrophils, while M0 macrophages showed significant negative correlations with resting mast cells and monocytes (**Figure 8C**). Further analysis showed that *ANXA1* was significantly associated with follicular helper T cells (*p* = 0.0097), resting mast cells (*p* = 0.0049), and M0 macrophages (*p* = 0.0038). Specifically, ANXA1 was positively correlated with resting mast cells (cor = 0.48) and negatively correlated with M0 macrophages (cor = -0.49) and follicular helper T cells (cor = -0.44). *CD36* was significantly positively correlated with resting mast cells (*p* = 0.0121, cor = 0.43), whereas *MICB* exhibited a significant negative correlation with M0 macrophages (*p* = 0.0297, cor = -0.38) (**Figure 8D**).

### 3.8 Regulatory network of biomarkers

**Figure 9:**
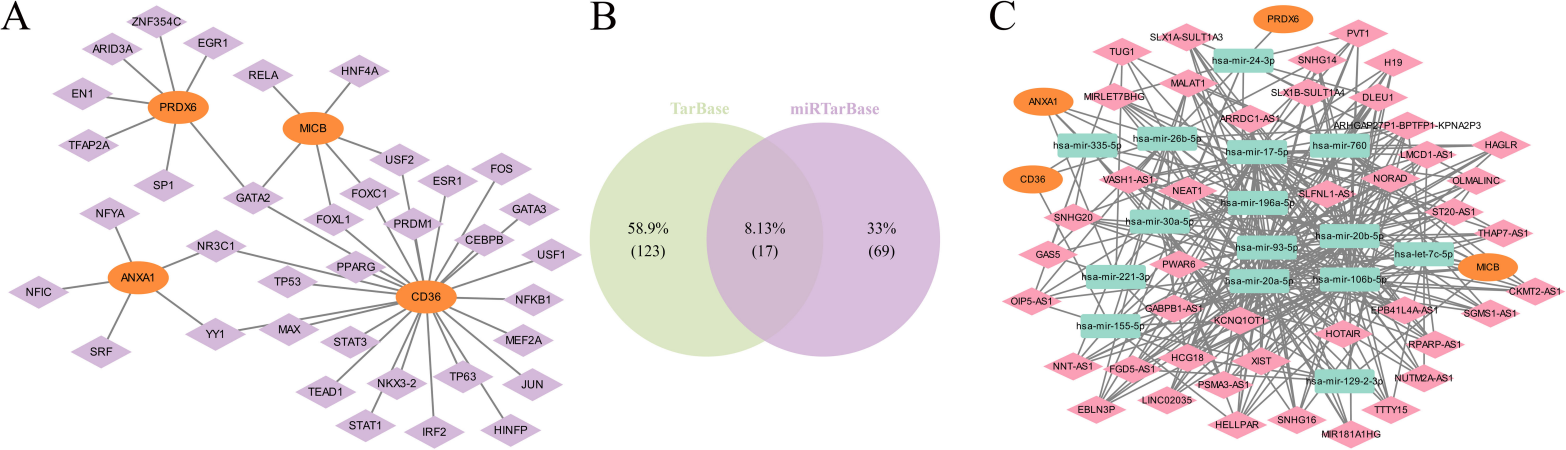
Regulatory network of biomarkers. **(A)** Network of TFs and biomarkers (TFs in purple, biomarkers in orange). **(B)** miRNA-mRNA interaction pairs. **(C)** ceRNA regulatory network (biomarkers in orange, miRNAs in green, lncRNAs in pink).

To investigate the potential mechanisms of the biomarkers in ULM, 36 TFs were predicted. Among these, GATA2 was associated with *CD36*, *MICB*, and *PRDX6*, while NR3C1 and YY1 were linked to *CD36* and *ANXA1* (**Figure 9A**). Additionally, 17 miRNA-mRNA interaction pairs were identified from two databases (**Figure 9B**), and 324 lncRNAs were predicted. Ultimately, a ceRNA network was constructed, involving 4 mRNAs, 15 miRNAs, and 324 lncRNAs. *PRDX6* was regulated solely by hsa-mir-24-3p and various lncRNAs, including PVT1, OLMALINC, and VASH1-AS1. *ANXA1* and *CD36* were regulated by hsa-mir-335-5p and hsa-mir-26b-5p (**Figure 9C**).

### 3.9 Associated between biomarkers and uterine diseases

**Figure 10:**
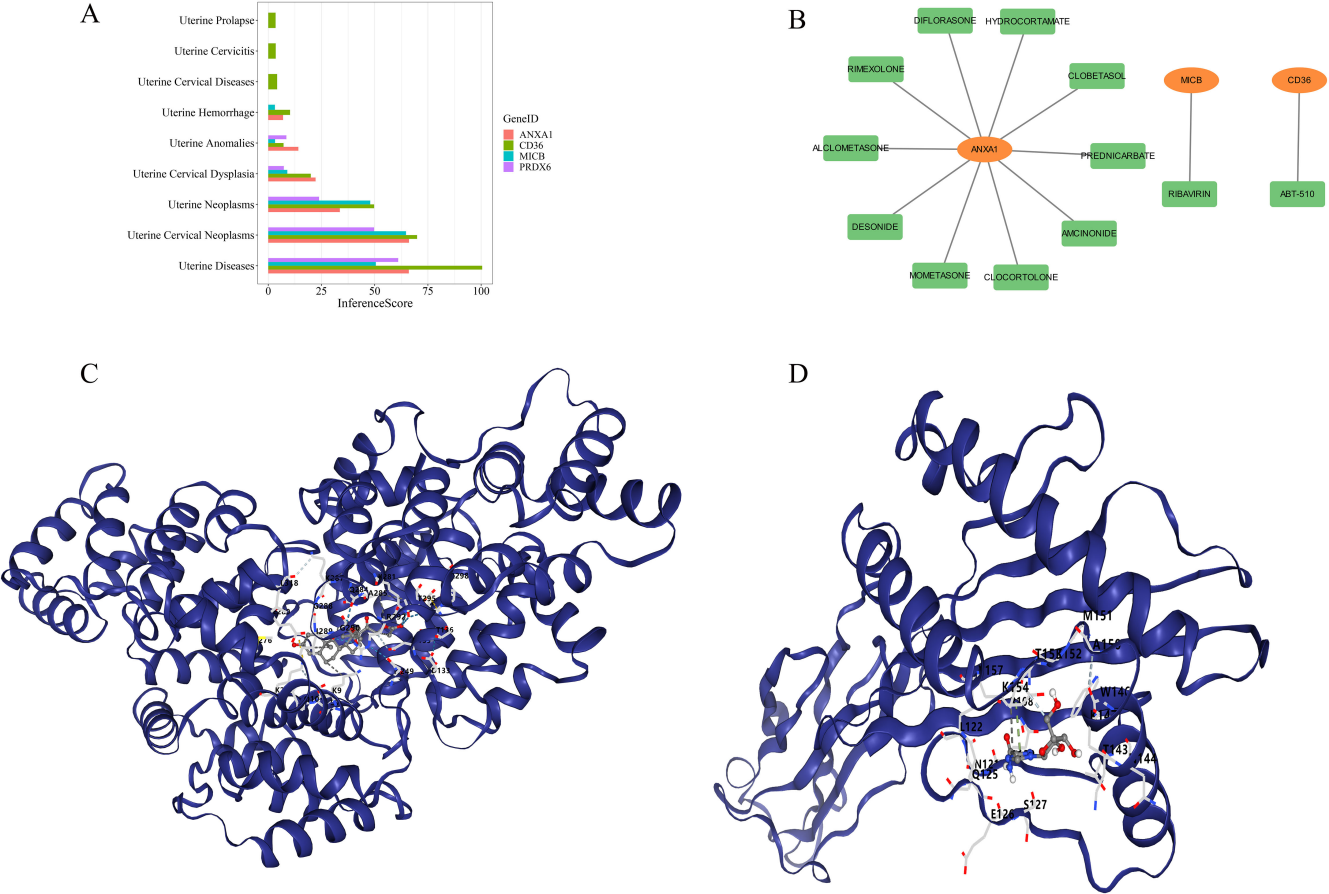
Gene-disease and gene-drug analysis. **(A)** Relationships between biomarkers and intrauterine diseases. **(B)** Relationships between biomarkers and small molecule drugs (biomarkers in orange, drugs in green). **(C)** Molecular docking between *ANXA1* and amcinonide. **(D)** Molecular docking between *MICB* and ribavirin.

The study also revealed associations between the 4 biomarkers and various uterine diseases. *CD36* was exclusively linked to uterine prolapse, uterine cervicitis, and uterine cervical diseases, while the other biomarkers were associated with conditions such as uterine hemorrhage, uterine anomalies, uterine cervical dysplasia, uterine neoplasms, and uterine cervical neoplasms (**Figure 10A**). Small-molecule drugs corresponding to the biomarkers were predicted as well. *ANXA1* exhibited the strongest interaction with amcinonide (interaction score = 1.77), *CD36* had a notable interaction with ABT-510 (interaction score = 15.46), and *MICB* interacted with ribavirin (interaction score = 1.93) (**Table 3**). A gene-drug interaction network was then established (**Figure 10B**). Subsequently, molecular docking was performed between the biomarkers and the drugs with the highest interaction scores. *ANXA1* and amcinonide had the lowest binding energy of -8.6 kcal/mol (**Figure 10C**), while *MICB* and ribavirin exhibited a binding energy of -6.2 kcal/mol (**Figure 10D**), indicating strong binding affinities.

**Table 3:** Correspondences between hub genes and drugs.

Gene	Drug	Query Score	Interaction Score
*ANXA1*	AMCINONIDE	2.46	1.77
*ANXA1*	MOMETASONE	2.15	1.55
*ANXA1*	HYDROCORTAMATE	2.15	1.55
*ANXA1*	CLOCORTOLONE	1.43	1.03
*ANXA1*	DIFLORASONE	1.43	1.03
*ANXA1*	ALCLOMETASONE	1.43	1.03
*ANXA1*	DESONIDE	1.43	1.03
*ANXA1*	CLOBETASOL	1.08	0.77
*ANXA1*	PREDNICARBATE	1.08	0.77
*ANXA1*	RIMEXOLONE	0.86	0.62
*CD36*	ABT-510	1.08	15.46
*MICB*	RIBAVIRIN	0.13	1.93

### 3.10 Expression of biomarkers

**Figure 11:**
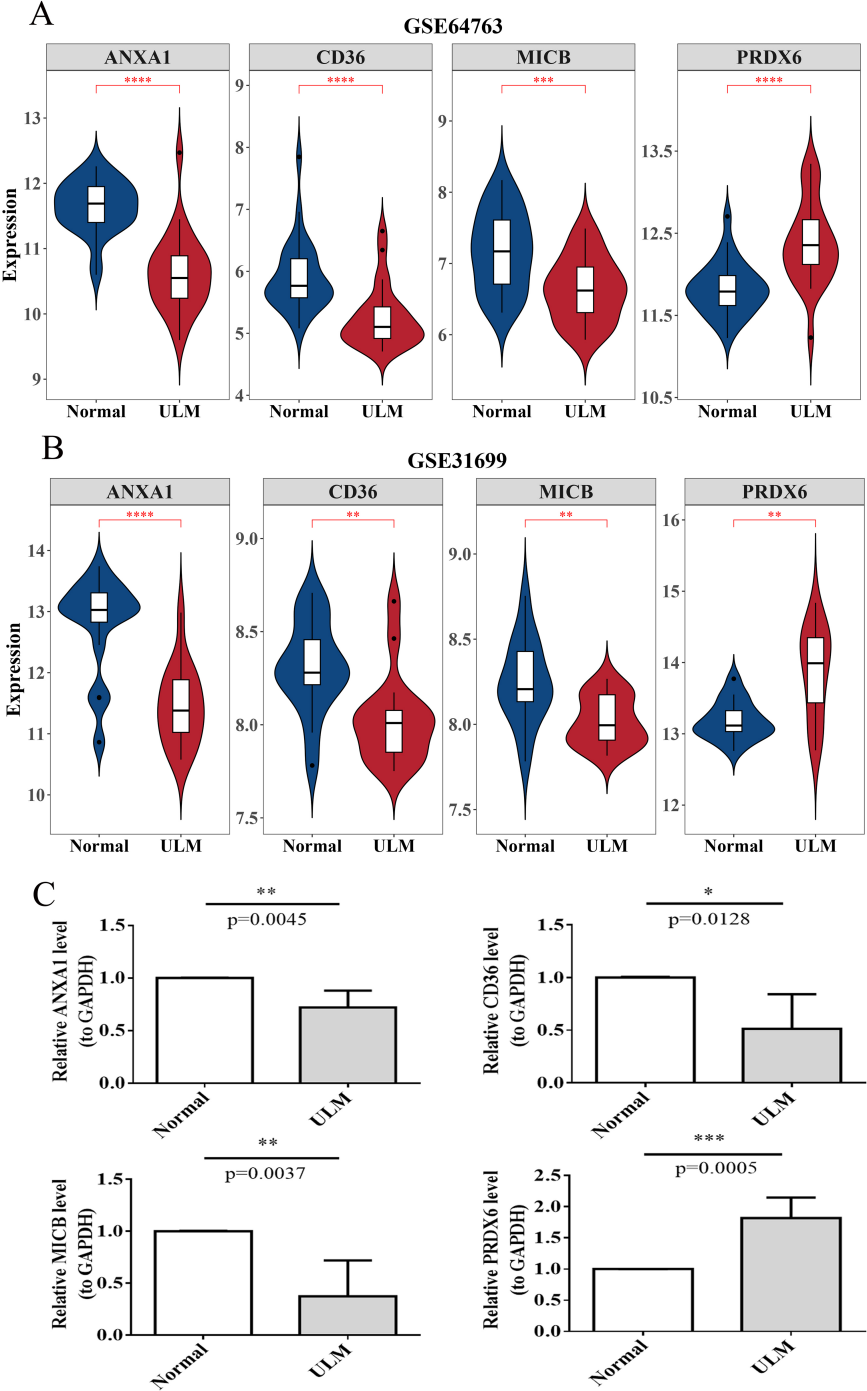
Biomarker expression analysis. **(A)** Violin plots of biomarker expression in the training set (GSE64763). **(B)** Violin plots of biomarker expression in the validation set (GSE31699). **(C)** RT-qPCR results for biomarkers in clinical samples. *p < 0.05; **p < 0.01; ***p < 0.001; ****p < 0.0001.

In terms of expression, the biomarkers showed significant differential expression in the training set. Specifically, *ANXA1*, *CD36*, and *MICB* were markedly downregulated in ULM samples, whereas *PRDX6* was upregulated (**Figure 11A**). These expression trends were consistent in both the validation set and RT-qPCR results, confirming the findings from the training set (**Figures 11B, C**).

## 4 Discussion

ULM is the most prevalent benign tumor in the female reproductive system, often asymptomatic and incidentally detected during routine medical examinations. Although the exact etiology of ULMs remains unclear and specific tumor markers for diagnosis are lacking (36), growing evidence implicates oxidative stress as a key factor in their development and progression (11, 37). Oxidative stress can induce abnormal proliferation of ULM cells, promote DNA synthesis, and alter cell cycle dynamics, thereby driving tumor growth (38–40). Additionally, it influences cell apoptosis, inflammatory responses, and angiogenesis, further exacerbating ULM pathogenesis (10, 41–43). However, the precise roles of oxidative stress-related genes in ULMs remain insufficiently understood. In this study, transcriptional datasets from the GEO database were analyzed, identifying four biomarkers (*ANXA1*, *CD36*, *MICB*, and *PRDX6*) associated with oxidative stress in ULMs through differential analysis, WGCNA, and MR. MR analysis revealed that these biomarkers exert significant effects on ULM. ROC curve analysis further underscored their diagnostic potential for ULMs. Additionally, a predictive alignment chart incorporating these biomarkers was developed, effectively forecasting ULM risk.

In the NCBI (National Center for Biotechnology Information), there are more than 10 datasets on ULM. These data sets are used in different studies for different purposes and sample characteristics. For example, the study by José A Castro-Martínez et al. (44) used two datasets, GSE12814 and GSE23112. GSE23112 focused on microRNA expression, while GSE12814 was associated with molecular signatures of the del(7q) UL subgroup. Through comprehensive transcriptome analysis, this study provides an in-depth understanding of the molecular mechanisms of uterine diseases and provides scientific support for future diagnosis and treatment strategies. Another study (45) combined four datasets, GSE64763, GSE45188, GSE30673, and GSE593, to deepen our understanding of the pathogenesis of ULM and to identify genes involved in the development of ULM. Among them, GSE64763 is consistent with the one selected in this study; GSE45188 showed the DNA methylation profile of myometria in uterine fibroids and non-fibroids. GSE30673 focuses on MED12 mutation; GSE593 contained genome-wide expression profiles of 5 uterine fibroids and 5 normal uterine tissues. Compared with other datasets, we finally decided to use the two datasets GSE64763 and GSE31699 after comprehensively considering factors such as research design, requirement matching degree and sample size.

*ANXA1*, a 37-kDa protein belonging to the annexin A family, plays multifunctional roles in both innate and adaptive immunity (46). In recent years, the role of *ANXA1* in tumors has garnered increased attention (47), with studies showing its expression varies by tissue type (48). For instance, *ANXA1* is overexpressed in melanoma, hepatocellular carcinoma, gastric cancer, and lung cancer (49), while its expression is diminished in prostate and esophageal cancers (50). Jamaluddin et al. conducted genetic and proteomic analyses on ULM and adjacent normal muscle tissues to characterize the expression patterns of extracellular matrix (ECM) proteins in MED12 mutation-positive and -negative ULMs. Among ECM-related proteins, *ANXA1* was found to be downregulated in ULMs of various sizes (51), aligning with the findings of this study and suggesting potential therapeutic targets.

MHC Class I polypeptide-related sequence B (*MICB*), a non-classical HLA-I gene within the human major histocompatibility complex, is highly polymorphic, with 40 polymorphic sites and 26 protein variants identified to date (52–54). *MICB* is a stress-inducible protein primarily expressed on cell membranes, acting as a ligand for NK cell receptors (53). Its surface expression is minimal in normal cells but markedly upregulated in tumor cells or cells infected with viruses (55). This study is the first to implicate *MICB* in ULM, laying the groundwork for future investigations. However, further research is needed to elucidate the underlying mechanisms of this interaction.

*CD36*, a transmembrane protein and member of the class B scavenger receptor family, interacts with other transmembrane proteins on the cell surface to mediate ligand binding and signal transduction (56). It is widely expressed on the surface of microvascular endothelial cells and plays a pivotal role in regulating endothelial cell function (57). In tumor microvasculature, *CD36* binds to thrombospondin-1 (TSP-1), mediating endothelial cell apoptosis within tumor vasculature (58). Knapp et al., in their study on energy substrate transport proteins in ULM, discovered that *CD36* expression was reduced in patients with ULM compared to matched healthy muscle layers (59). These findings align with our study results.

*PRDX6*, a peroxidase enzyme composed of 224 amino acids, is expressed in various tissues and organs. Recent research has highlighted its significant role in cancer. *PRDX6* is notably overexpressed in cervical cancer tissues, where its overexpression promotes proliferation, migration, and invasion of cancer cells while inhibiting apoptosis (60). Our findings similarly indicate elevated PRDX6 expression in ULM. Research has demonstrated that *PRDX6* knockout in HepG2 cells results in slowed cell division, mitochondrial dysfunction, and cell cycle arrest at the G2/M phase (61).

GSEA of *ANXA1*, *MICB*, *CD36*, and *PRDX6* revealed common enrichment in signaling pathways, including chemokine signaling and cytokine-cytokine receptor interactions. These pathways are hypothesized to play a role in ULM development. Xia et al., in their comprehensive analysis of four ULM-related mRNA datasets, suggested that extracellular matrix-receptor interactions could lead to abnormal extracellular matrix formation in ULM. Additionally, focal adhesion and cell adhesion molecules were implicated in ULM pathogenesis. Xia et al. further proposed that chemokine signaling and cytokine-cytokine receptor interactions are likely involved in ULM progression (45). Prates et al. demonstrated that *ANXA1* influences cervical cancer development by activating the transcriptional expression of formyl peptide receptors (FPRs) and the inhibitor of DNA binding 1 (ID1) (62). *FPR* plays a central role in the chemokine signaling pathway, while ID1, a DNA transcription regulator, is closely linked to DNA replication. Our findings are consistent with their results, reinforcing the involvement of these pathways in ULM development.

Through in-depth analysis of the functional and pathway information enriched by GO and KEGG, we found several clues closely related to the pathogenesis or related hypotheses of ULM. Studies have shown that ROS, as a pro-inflammatory mediator, can regulate cell proliferation and is known to activate the MAPK/ERK pathway in endometriosis (63), suggesting that ROS may also be involved in the pathological process of ULM, although this hypothesis needs to be further verified. Notably, ganirelix as a potential therapeutic agent has demonstrated significant toxicity to ULM cells, suggesting that it may induce tumor reduction in a broad patient population (64). In addition, chronic inflammation caused by visceral fat plays a key role in cell differentiation and proliferation, which is a necessary condition for the onset of ULM. Increased visceral fat deposits may alter cholesterol composition, thereby increasing the risk of subclinical atherosclerosis in patients with ULM (65). Fibromodulin (FMOD), as an important component of the extracellular matrix, not only plays a role in structure, but also is regarded as a novel tumor-associated antigen for a variety of malignant tumors, including ULM, and has the potential as a biomarker for cancer diagnosis and treatment (66). Relaxin has also been suggested to be involved in the pathogenesis of ULM, providing a new perspective for disease research (67). At the same time, TNF-α up-regulates MMP-2 expression and promotes cell migration by activating extracellular signal-regulated kinase (ERK) signaling pathway in uterine fibroid smooth muscle cells, which provides a theoretical basis for the development of ULM treatment strategies targeting TNF-α and MMPs inhibitors (68).

Immunoreactive cells, including lymphocytes, macrophages, granulocytes, and dendritic cells, are crucial participants in immune responses. Recent studies suggest that the composition and abundance of these cells may influence the development and progression of uterine ULMs (69). Using the CIBERSORT algorithm, we analyzed the immunoreactive cell infiltration characteristics in ULM versus normal tissues, revealing significant differences in five types of immunoreactive cells between the two groups. Supporting our findings, Zannotti et al. reported a greater abundance of macrophages within and around ULMs compared to distant myometrial tissue (69). The dysregulation of macrophage proliferation, infiltration, and accumulation contributes to pathological fibrosis and uncontrolled tissue repair, with key roles played by factors such as MCP-1, GM-CSF, TGF-β, and TNF-α. Similarly, Protic et al. observed a higher density of CD68-positive macrophages in ULMs and adjacent myometrium compared to distant myometrium (70). In this context, TNF-α secreted by macrophages may enhance activin A mRNA expression in ULM cells, potentially leading to excessive extracellular matrix production, tissue remodeling, and tumor growth. However, a clinical study by Liu et al. found no significant differences in MCT-positive mast cells or CD45-positive leukocytes between ULM and normal tissues in premenopausal women (71).

In another study, Wang et al. identified significant overexpression of miR-30a, miR-34, and miR-24, in addition to Let-7, in ULM. These miRNAs may inhibit ULM proliferation and differentiation by suppressing *HMGA2a*, acting as protective factors against the overexpression of oncogenes such as *RAS* and *MYC (* 72). Extensive research has also explored miR-196a’s role in various cancers, showing its involvement in key biological processes related to tumorigenesis. Hu et al. demonstrated that miR-196a regulates the proliferation, invasion, and migration of esophageal squamous cell carcinoma by targeting *ANXA1* (73). In breast cancer, miR-196a expression correlates with certain *HOXC* genes involved in tumor progression (74), while in cervical cancer, it targets *netrin 4*, influencing cell proliferation and migration (75). Although ULMs are benign, their abnormal cellular proliferation mirrors that of malignant tumors, suggesting that miR-196a may play a similar role in ULM pathogenesis. Furthermore, miR-155-5p exhibits dual roles, acting as an oncogene or tumor suppressor depending on the cellular environment and cancer type. Research by Navarro et al. indicated that miR-155-5p is implicated in the inflammatory processes often associated with ULMs (76).

Our research identified a strong binding affinity between *ANXA1* and amcinonide, as well as between *MICB* and ribavirin. Previous studies have demonstrated that amcinonide, an anti-inflammatory agent with affinity for glucocorticoid receptors, can induce depigmentation in both black and albino mice. It significantly reduces the number of DOPA-positive epidermal melanocytes in these animals (77). Ribavirin, a broad-spectrum antiviral agent effective against HCV, HIV, and RSV, has been shown to decrease activin A levels while increasing follicle-stimulating hormone concentrations in the serum and liver of Wistar rats (78). The drug-binding analysis in this study suggests that amcinonide and ribavirin may serve as potential therapeutic agents for ULM, interacting with *ANXA1* and *MICB*, respectively. While these findings hold promise for therapeutic interventions in ULM, further investigation into the precise mechanisms of action is necessary.

In conclusion, this study identified oxidative stress-related biomarkers in ULM and provided a comprehensive analysis of drug networks and immune cell infiltration, shedding light on the molecular mechanisms of these biomarkers in ULM. The findings offer potential new avenues for ULM treatment.

However, several limitations should be noted. The relatively small sample size used in this study may have introduced bias and affected both the statistical power and the biological relevance of the results. Future studies will aim to include larger sample sizes to further validate the accuracy of these biomarkers and their association with disease. At the same time, we will also consider implementing cross-validation strategies on a broader dataset to further consolidate and extend our research results. Additionally, the study focused exclusively on gene expression levels, which do not necessarily reflect direct biological effects. To validate these findings, further animal and clinical studies employing different experimental approaches will be required.

## Data Availability

The original contributions presented in the study are included in the article/**Supplementary Material**. Further inquiries can be directed to the corresponding author/s.

## Supplementary material

Supplementary Figure 1 Experimental technical roadmap illustrating study workflow.

Supplementary Figure 2 Sample clustering and ULM gene feature heatmap using WGCNA.

Supplementary Table 1 Primer sequences used in the study.

Supplementary Table 2 F-statistic information for SNP.
