# Supplementary figures and images for: Identification of oxidative stress-related biomarkers in uterine leiomyoma: a transcriptome-combined Mendelian randomization analysis
Source: Front Endocrinol (Lausanne). 2024 Nov 21;15:1373011. doi: 10.3389/fendo.2024.1373011 (PMC11617171; doi:10.3389/fendo.2024.1373011)

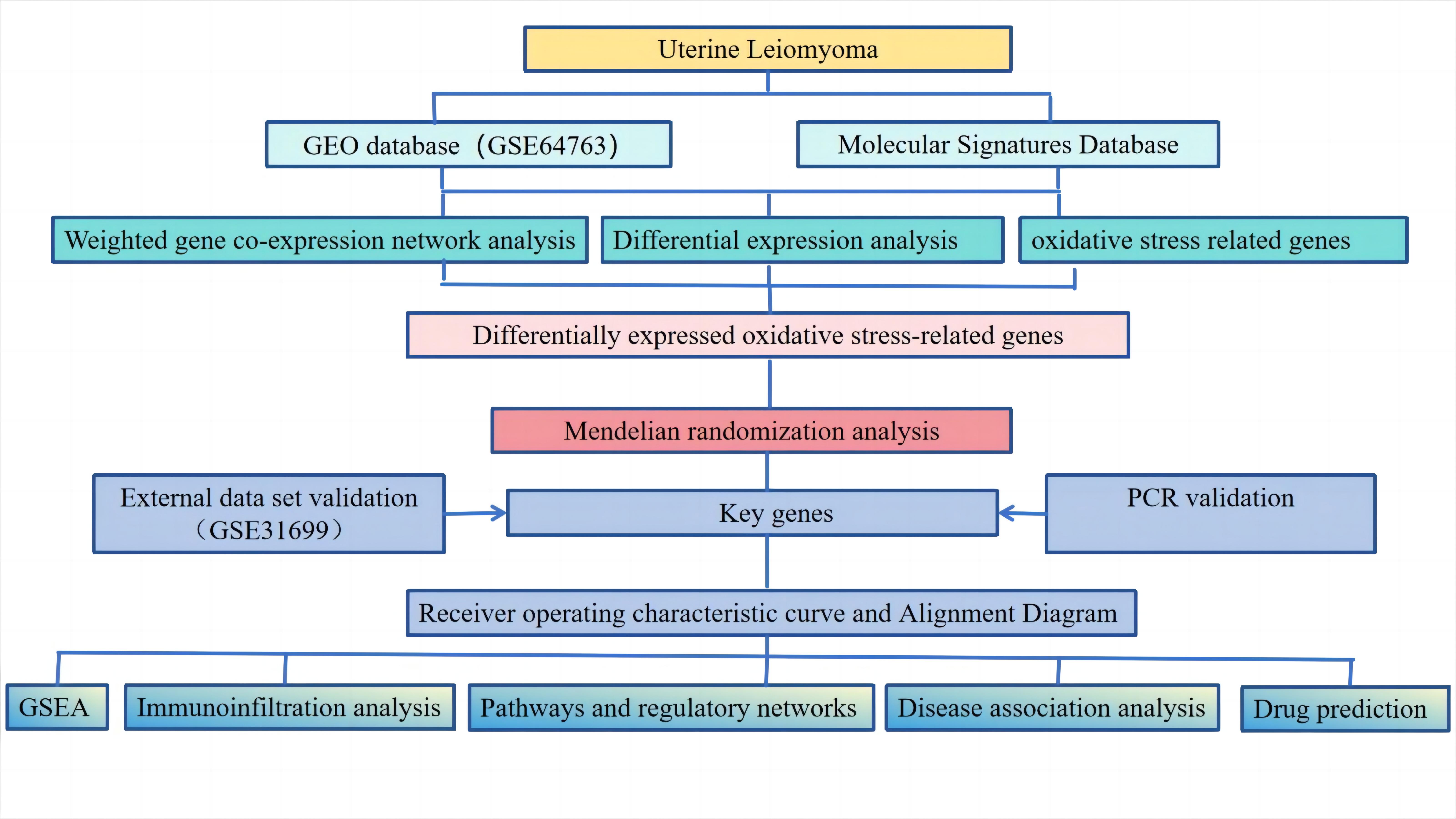

Supplement: Supplementary Figure 1 — Experimental technical roadmap illustrating study workflow. [file Image1.jpeg]

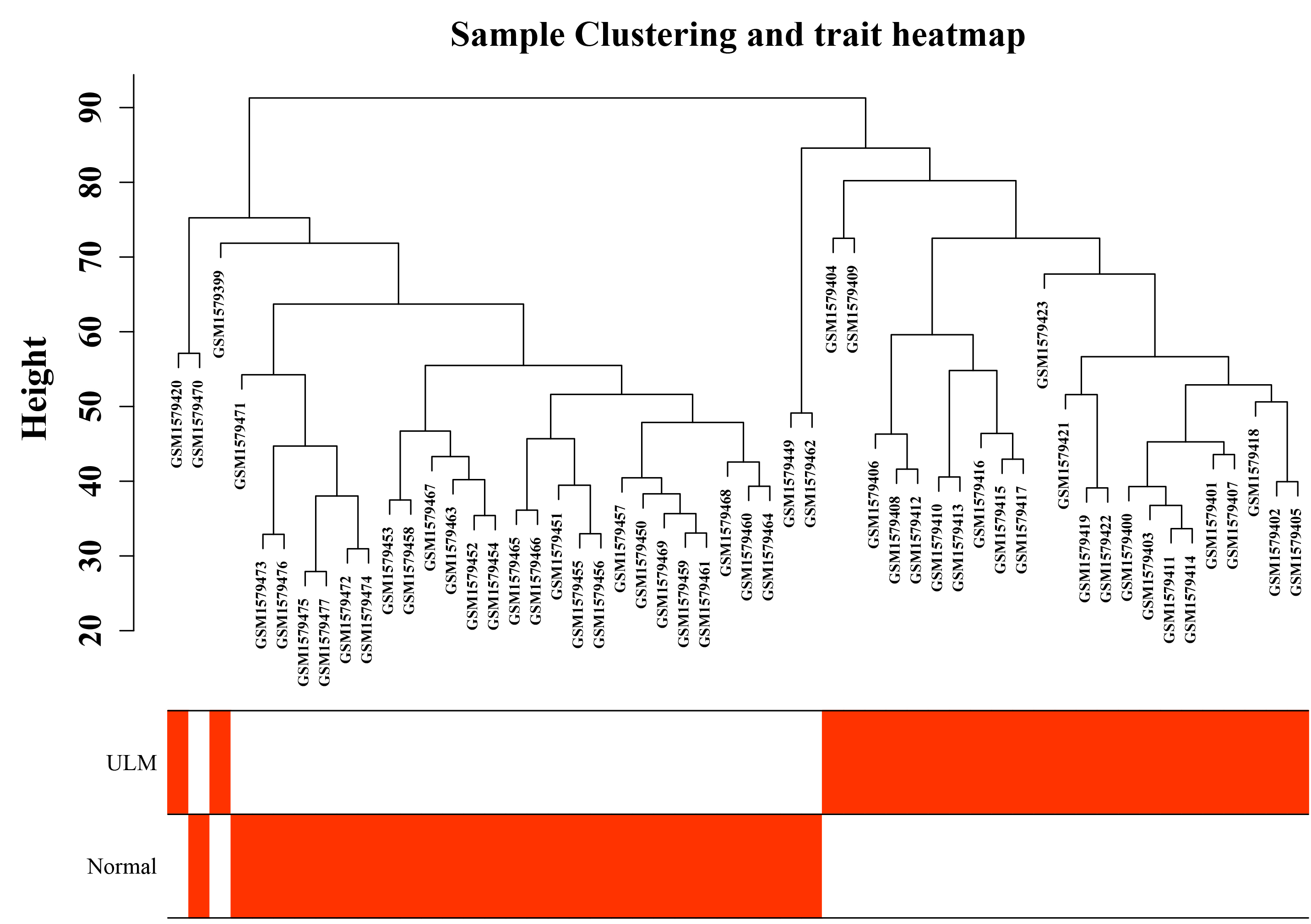

Supplement: Supplementary Figure 2 — Sample clustering and ULM gene feature heatmap using WGCNA. [file Image2.jpeg]
